# Positive Feedback Regulation between Phospholipase D and Wnt Signaling Promotes Wnt-Driven Anchorage-Independent Growth of Colorectal Cancer Cells

**DOI:** 10.1371/journal.pone.0012109

**Published:** 2010-08-12

**Authors:** Dong Woo Kang, Do Sik Min

**Affiliations:** Department of Molecular Biology, College of Natural Science, Pusan National University, Busan, Republic of Korea; Ohio State University, United States of America

## Abstract

**Background:**

Aberrant activation of the canonical Wnt/β-catenin pathway occurs in almost all colorectal cancers and contributes to their growth, invasion and survival. Phopholipase D (PLD) has been implicated in progression of colorectal carcinoma However, an understanding of the targets and regulation of this important pathway remains incomplete and besides, relationship between Wnt signaling and PLD is not known.

**Methodology/Principal Findings:**

Here, we demonstrate that PLD isozymes, PLD1 and PLD2 are direct targets and positive feedback regulators of the Wnt/β-catenin signaling. Wnt3a and Wnt mimetics significantly enhanced the expression of PLDs at a transcriptional level in HCT116 colorectal cancer cells, whereas silencing of β-catenin gene expression or utilization of a dominant negative form of T cell factor-4 (TCF-4) inhibited expression of PLDs. Moreover, both PLD1 and PLD2 were highly induced in colon, liver and stomach tissues of mice after injection of LiCl, a Wnt mimetic. Wnt3a stimulated formation of the β-catenin/TCF complexes to two functional TCF-4-binding elements within the PLD2 promoter as assessed by chromatin immunoprecipitation assay. Suppressing PLD using gene silencing or selective inhibitor blocked the ability of β-catenin to transcriptionally activate PLD and other Wnt target genes by preventing formation of the β-catenin/TCF-4 complex, whereas tactics to elevate intracellular levels of phosphatidic acid, the product of PLD activity, enhanced these effects. Here we show that PLD is necessary for Wnt3a-driven invasion and anchorage-independent growth of colon cancer cells.

**Conclusion/Significance:**

PLD isozyme acts as a novel transcriptional target and positive feedback regulator of Wnt signaling, and then promotes Wnt-driven anchorage-independent growth of colorectal cancer cells. We propose that therapeutic interventions targeting PLD may confer a clinical benefit in Wnt/β-catenin-driven malignancies.

## Introduction

Colorectal cancer is one of the most common malignancies, occurring in a significant percentage of the population. More than 80% of sporadic and hereditary colorectal cancers may be caused by aberrations in the Wnt/β-catenin signaling pathway [1]–[3]. Thus, alterations in the Wnt/β-catenin pathway define a key event in the pathogenesis of colon cancer. β-Catenin is a transcriptional coactivator of T cell factor (TCF)/lymphoid enhancer factor (Lef) transcription factors. β-catenin stability is regulated by a multiprotein complex that includes adenomatous polyposis coli (APC), glycogen synthase kinase 3β (GSK3β), and axin. Phosphorylation of β-catenin by GSK3β targets β-catenin to ubiquitination and proteasome degradation [4]. Thus, activation of the pathway represses β-catenin degradation, resulting in nuclear accumulation of β-catenin. In the nucleus, accumulation of TCF/β-catenin leads to transcriptional activation of multiple target genes, which can then contribute to development of cancer [5], [6]. Thus, identification of direct targets of the Wnt/β-catenin signaling pathway is potentially important to understanding the central role of the Wnt/β-catenin/TCF dependent canonical pathway in tumorigenesis.

Phospholipase D (PLD) catalyzes hydrolysis of phosphatidylcholine (PC) to generate phosphatidic acid (PA), which acts as a second messenger in many physiological responses [7]. Two mammalian PC-specific PLD isozymes designated as PLD1 and PLD2 have been cloned. PLD has emerged as a critical regulator of cell proliferation and survival whose dysregulation occurs during development of a variety of human tumors [8]. Elevated expression of PLD1 and PLD2 has been reported in colorectal cancer tissues [9]; in particular, PLD2 expression level and its association with clinicopathological features have recently been investigated in colorectal carcinoma [10]. Expression levels of PLD2 correlate significantly with tumor size and survival of patients with colorectal carcinoma [10]. The PLD2 point mutation has also been found in breast cancer [11]. Cells overexpressing PLD isozyme enhance matrix metalloproteinase-2 expression and tumor cell invasion and form metastases in syngeneic mice [12], [13]. These findings suggest that PLD plays an important role in progression of colorectal carcinoma, and could be a target for cancer therapy. We have recently reported on significant co-overexpression of PLD isozymes with β-catenin in human colorectal cancer [14]. Using two RNA interference (RNAi)-based loss-of-function screens, the oncogenes that modulate β-catenin-dependent transcription and regulate colon cancer cell proliferation have been identified [15].

Among one of the genes identified in this screen was PLD1, and suppression of PLD1 significantly inhibited both β-catenin transcriptional activity and colon cancer cell proliferation. In the present study, we demonstrate the action of PLD isozymes as novel targets and positive feedback regulators of Wnt signaling. Thus, identification of a Wnt-β-catenin-TCF-regulated PLD axis provides new mechanistic insights into cancer.

## Materials and Methods

### Cell lines and reagents

Human colorectal cancer cells (HCT116, HCA-7, Colo-741, RKO) and breast cancer cells (HS578T) were purchased from ATCC (Manassas, VA) and were grown according to standard protocols. Purified recombinant Wnt3a was purchased from R&D Systems Inc. BIO was obtained from Calbiochem. LiCl, 1- or 3-butanol, dioctanoyl PA, and 1-propranolol were purchased from Sigma-Aldrich. PLD1 and PLD2 selective inhibitors were purchased from Cayman chemical. Dual luciferase assay kits were purchased from Promega.

### Plasmids and small interfering RNA

Human PLD1 (pGL4-PLD1 Luc) and PLD2 (pGL4-PLD2 Luc) promoter reporter plasmids contain −1.9 kb (−1930/+1) and 2.6 kb (−2180/+491) of upstream 5′-flanking DNA linked to luciferase reporter genes, respectively, and have been described elsewhere [14]. We used the promoter of hPLD1 (pGL4-PLD1; −1930/+1), transcribed from exon 2, among two alternate transcripts of PLD1 to be transcribed at two different transcription sites (exon 1 and exon 2). The PCR-based method was used for cloning of serially deleted PLD2 promoter constructs into the pGL4-14b reporter vector at the Kpn I and Bgl II site. The sequence of the oligonucleotides used as primers in PCR amplifications is shown in Table S1.

Mutations of TCF-4 binding elements on the PLD2 promoter were generated using the Quick Change Site-Directed Mutagenesis Kit, according to the manufacturer's recommendations (Stratagene; Table S2). TOPflash and FOPflash luciferase plasmids containing multiple copies of TCF/LEF response elements were used for measurement of TCF activity. Dominant-negative TCF-4 (ΔN30 TCF-4) was kindly provided by Dr. Tesshi Yamada (National Cancer Center Research Institute). Constitutive active mutant of β-catenin (S37A β-catenin) and wild type TCF-4 expression vectors were kindly provided by Dr. Bert Vogelstein (Johns Hopkins University). shRNA for β-catenin was kindly provided by Dr. H. Clevers (Utrecht, The. Netherlands). SiRNAs for control, PLD1, and PLD2 were purchased from Dharmacon Research Inc (Lafayette, Colo). siRNA sequences for PLDs are as follows: human PLD1 (nucleotides, 1571 to 1591, AAGGUGGGACGACAAUGAGCA) and human PLD2 (nucleotides, 1378 to 1396, ACAUAAAGGUGAUGCGUCA).

### Transient Transfection and Reporter Gene Assay

Following the manufacturer's instructions, expression plasmids or siRNA were transiently transfected into cells using LipofectAmine Plus (Invitrogen) or Polyfect (Qiagen) reagents. Transfection and luciferase assays were performed as previously described [16]. Relative luciferase activity was obtained by normalization of the activity of firefly luciferase against activity of the internal control *renilla* luciferase.

### Immunoprecipitation and Western Blotting

Cell lysates were analyzed for immunoprecipitation and/or immunoblot, as previously described [17]. Anti-α-tubulin (Sigma, MO), anti-vimentin (Sigma, MO), anti-c-Myc (Santa Cruz, CA), anti-β-catenin (Santa Cruz, CA), anti-NOS2 (Santa Cruz, CA), anti-TCF-4 (Santa Cruz, CA), anti-β-catenin (BD Biosciences, CA), anti-cycinD1 (BD Biosciences, CA), and anti-phospho-GSK3β antibody (Cell signaling, MA) were purchased. The polyclonal anti-PLD antibody that recognizes both PLD1 and PLD2 was generated as previously described [18].

### PLD activity assay

PLD activity was assessed by measurement of the formation of [^3^H] phosphatidylbutanol, the product of PLD-mediated transphosphatidylation, in the presence of 1-butanol, as previously described [17].

### Quantitative RT-PCR

Total RNA (1 µg) was pretreated with DNase and used for reverse transcription with M-MLV reverse transcriptase (Invitrogen). Real-time Q-PCR was performed on a Rotor-Gene RG-6000A apparatus (Corbett Research): for 44 cycles of 94°C for 10 sec, 60°C for 10 sec, and 72°C for 15 sec. Reactions (20 µl) included 2 µl of cDNA, target-specific primers, and the Quantitect SYBR green PCR kit (QIAGEN). The temperature range for analysis of melting curves was 55°C to 99°C over 30 sec. Three independent experiments were performed for each reaction in triplicate. All data were normalized with GAPDH gene expression values. See Table S3 for Q-RT-PCR primer sequences.

### Animal and tissue preparation

Mice were provided with standard maintenance and a diet from Dae Han Bio Link (Seoul, Korea). Animal studies were approved and performed under guidelines of the Institute of Health Guidelines for the Institutional Review Board of Pusan National University (Busan, Korea). Twelve week-old male FVB mice received intravenous injection with LiCl once each day for 2 days at dose of 5 mg/kg. The control group was injected with the same volume of phosphate-buffered saline (PBS). Forty-eight hrs after LiCl injection, animals (n = 3/group) were deeply anesthetized with diethyl ether and decapitated, and the tissues were frozen immediately in LN_2_ and stored at −70°C for immunoblotting. Mice (n = 3/group) also were anesthetized and then perfused intracardially with 4% paraformaldehyde in PBS containing 0.34% L-lysine (Sigma). Following fixative perfusion, the tissues were removed, placed in 4% paraformaldehyde solution at 4°C overnight, and then transferred to a 30% sucrose solution. The tissues were processed by coronal cryostat sectioning in Dulbecco's PBS solution containing 0.1% sodium azide using a freezing microtome (MICROM, Germany).

### Immunohistochemistry

Paraformaldehyde-fixed 4 µm paraffin sections of colon tissues were autoclaved in 10 mM sodium citrate buffer (pH 6.0). After blocking with 5% BSA and 1% normal goat serum in PBS, sections were incubated with anti-β-catenin (BD transduction) and PLD antibodies overnight at 4°C, followed by washing with PBS. Subsequently, sections were incubated with Alexa fluor 488 or Alexa fluor 555-conjugated IgG secondary antibody (Santa cruz, 1:200) at room temperature for 1 h, followed by washing with PBS. After counterstaing with DAPI, and slides were mounted in Permount^®^ (Fisher Scientific, USA). Two-color fluorescent image for anti-PLD and β-catenin antibody staining were collected on a Zeiss LSM 510 confocal microscope (Zeiss, Germany). Fluorescent images were analyzed using Zeiss LSM image browser software (Zeiss).

### Chromatin immunoprecipitation (ChIP) assay

ChIP experiments were performed as previously described [19], with minor modifications. HCT116 cells were used for crosslinking with 1% paraformaldehyde in phosphate-buffered saline (PBS) for 10 min. Cells were scraped and collected by centrifugation. Cells were lysed in lysis buffer (50 mM Hepes, pH 7.5, 140 mM NaCl, 1 mM EDTA, 1% Triton X-100, 0.1% deoxycholate, and 1.0 mM protease inhibitor cocktail) and sonicated for 20 sec 3 times. Normal mouse IgG, anti-β-catenin or anti-HDAC1 antibody was added and incubated for 6 h at 4°C. Immnunocomplexes were extracted 3 times with 1% SDS, 0.1 M NaHCO3, and crosslinking was reversed by incubation at 65°C overnight. The saved chromatin input fraction was also processed in the same manner. Samples were then digested with DNase- and RNase-free proteinase K at 50°C for 4 h, and extracted with phenol/chloroform/isoamylalcohol. DNA samples were purified with Qiagen clean-up columns. The PLD2 or NOS2 promoter region was analyzed by Q-RT-PCR using specific primers (Table S4).

### Cell Migration and Invasion Assays

Cells were added to Transwell membrane chambers (pore size, 12.0 µm; Corning). The number of cells that migrated through the membrane to the lower chamber was counted after 24 hours. For siRNA experiments, cells were seeded 24 hours after transfection with siRNAs, and migration assays were performed for another 24 hours. For the invasion assays, Matrigel (1:5; BD) was added to Transwell membrane chambers and incubated for 5 hours; cells were then seeded. Extent of migration and invasion were expressed as an average number of cells per microscopic field.

### Anchorage-independent growth assay

Anchorage independent growth was measured using the CytoSelect™ 96-Well *In Vitro* Tumor Sensitivity Assay (Cell Biolabs, CA), according to the manufacturer's specifications. Anchorage independent growth was examined in soft agar; 50 µl of base agar matrix was added to the bottom of each well of a 96-well plate. Once the agar was solid, 75 µl of cell suspension/soft agar matrix containing 3×10^3^ cells was layered onto the top, followed by 50 µl of 2× complete medium with Wnt3a and/or inhibitors. After 10 days of incubation, the agar matrix was solubilized, and 3-(4,5-dimethylthiazol-2-yl)-2,5-diphenyl tetrazolium bromide was added to each well. The absorbance produced by formation of insoluble formazan product by viable cells was recorded at 570 nm.

### Statistical analysis

All experiments were independently performed at least three times, with similar results. Data were analyzed by the Student's *t* test, and *P*<0.05 was considered statistically significant.

## Results

### Wnt signaling promotes expression of PLD isozymes

**Figure 1:**
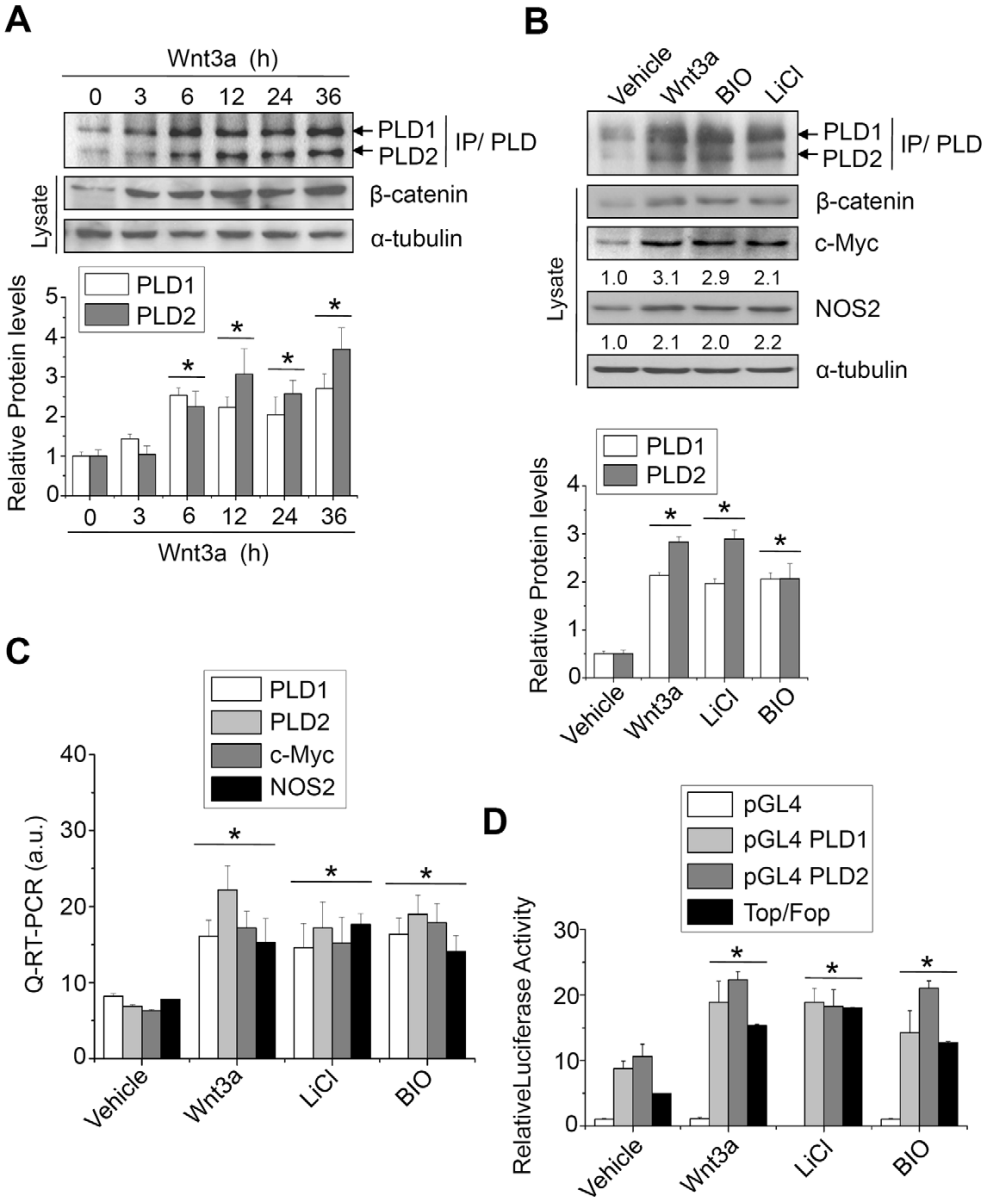
Wnt3a and GSK3β inhibitors increase expression of PLD isozymes in HCT116 colon cancer cells. (A) HCT116 cells were treated with purified recombinant Wnt3a (150 ng/ml) for the indicated times, and the lysates were immunoprecipitated and immunoblotted with antibody to PLD. β-catenin was analyzed by Western blotting using nuclear lysates (upper panel). Histograms show relative protein levels of PLD1 and PLD2, which are normalized to the corresponding α-tubulin values (lower panel). (B-C) HCT116 cells were treated with Wnt3a (150 ng/ml), LiCl (20 mM), or BIO (1 µM) for 24 h. (B) Protein level of PLDs was analyzed by immunoprecipitation and immunoblotting. c-Myc and NOS2 expression was analyzed by Western blotting (upper panel). Histograms show relative protein levels of PLD1 and PLD2, which are normalized to the corresponding α-tubulin values (lower panel). Data are representative of three independent experiments. (C) mRNA levels of the indicated genes were analyzed by Q-RT-PCR. *P<0.05 *versus* vehicle. (D) The indicated promoter reporter plasmids were transfected and treated with Wnt3a, LiCl, or BIO for 12 h; luciferase activity was then determined. *P<0.01 *versus* vehicle. Data represent the mean ± S.D. of three independent experiments.

To examine the question of whether or not Wnt signaling increases PLD expression, HCT116 colorectal cancer cells were exposed to Wnt3a or Wnt3a mimetics (Figure 1). As determined by immunoprecipitation and Western blot, expression of both PLD1 and PLD2 was enhanced in a time-dependent manner following exposure to purified recombinant Wnt3a (150 ng/ml) (Figure 1A, upper panel). Anti-PLD antibody was generated against C-terminal peptide of PLD1 in which 7 amino acids among 12 amino acids were the same with that of PLD2, and recognizes both PLD1 and PLD2 [18]. It was demonstrated that HCT116 cells have mutations in β-catenin at Ser-45 and that this Ser-45 mutant allele was not sufficient to support tumorigenesis of HCT116 cells [20]–[22]. Thus, it is suggested that the β-catenin pathway in HCT116 cells is not fully activated by the mutation, and therefore can be stimulated by additional extracellular messengers, such as Wnt signaling, resulting in further oncogenic transformation. We then investigated the question of whether or not Wnt signaling-induced PLD expression might be regulated at a transcriptional level. Wnt3a enhanced the mRNA levels of PLD1 and PLD2 in a time-dependent manner (Figure S1). To exclude that Wnt-dependent increase of PLD mRNA is only caused by changes in mRNA stability, we performed a mRNA decay assay using actinomycin D, which prevents transcription. The expression of PLD1 and PLD2 was increased over time in a comparable fashion between Wnt-treated and control cells as analyzed by Q-RT-PCR (Figure S2). Pretreatment with actinomycin D significantly suppressed both basal and Wnt3a-induced PLD mRNA levels (Figure S2). Thus, Wnt-dependent increase of PLD mRNA is indeed due to elevated transcription. In order to further strengthen our results, we determined the effect of blockade of GSK-3β on PLD expression. LiCl and BIO are established agonists that mimic the Wnt-signaling pathway, leading to activation and stabilization of β-catenin [23], [24]. As analyzed by immunoprecipitation and Western blot, LiCl (20 mM), BIO (1 µM), and Wnt3a (150 ng/ml) significantly increased the expression level of PLD isozymes (Figure 1B). Wnt mimetics also increased the protein level of β-catenin, as well as expression of c-Myc and NOS2, target genes of Wnt signaling. As analyzed by Q-RT-PCR, Wnt signaling markedly elevated expression levels of PLD mRNA (Figure 1C). Additionally, Wnt and its mimetics significantly enhanced the promoter activity of both PLD1 and PLD2 (Figure 1D); Wnt3a accompanied a significant increase in gene expression from a TCF/LEF specific luciferase reporter plasmid (TOPflash) used as a control. Moreover, we found that Wnt3a increased expression levels of PLD isozymes in various cancer cells, including colorectal cancer cells (HCA-7, RKO, Colo-741) and breast cancer cells (HS578T) (Figure S3). To observe the induction of PLD isozymes in response to Wnt signaling, we chose cancer cells in which the status of the Wnt pathway is normal. These cancer cells are known to express wild type APC and β-catenin [25]–[29]. Thus, it is suggested that Wnt signaling-induced PLD expression might be a general phenomenon. Upregulation of PLDs expression by Wnt3a and Wnt mimetics strongly implicated a central role for inhibition of GSK3β and the canonical Wnt signalling pathway in Wnt-mediated effects. Taken together, these results indicate that induction of the PLD isozyme as a novel target of Wnt signaling is regulated at both the transcriptional and post-transcriptional levels.

### LiCl induces expression of PLD isozymes *in vivo*

**Figure 2:**
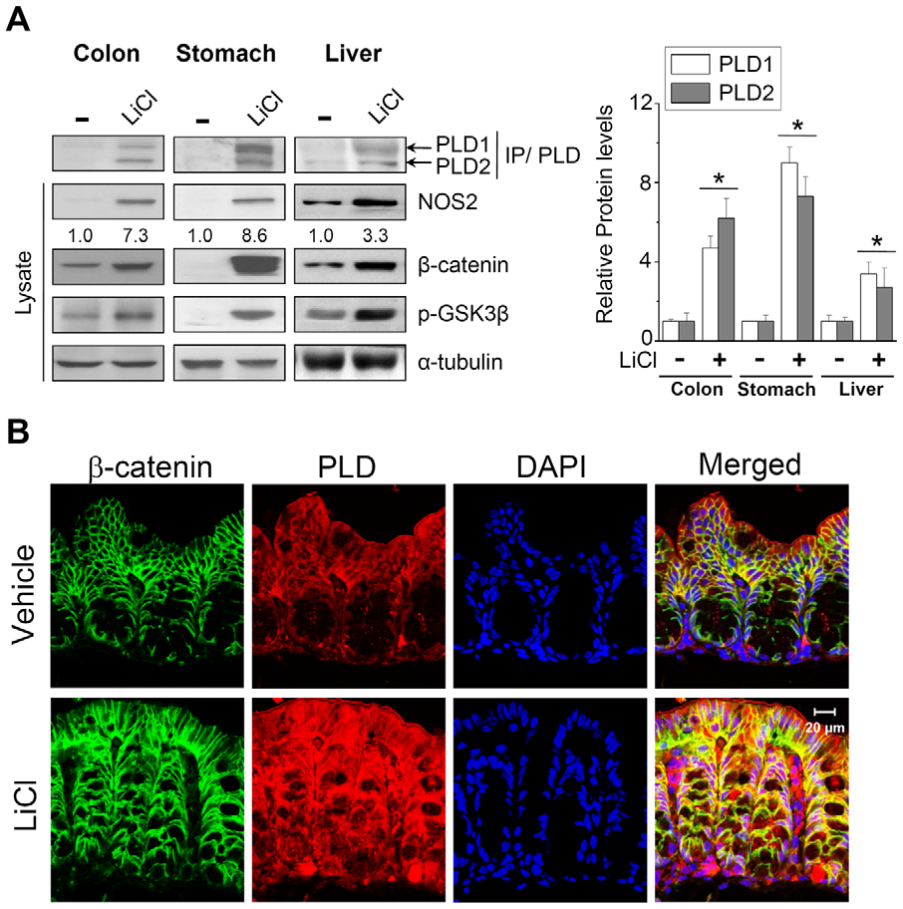
LiCl increases expression of PLD isozymes *in vivo*. (A) Mice were intravenously injected with LiCl, as described in “Materials and Methods”. Lysates from various tissues were immunoprecipitated and immunoblotted with antibody to PLD recognizing both PLD1 and PLD2 (left panel). Protein levels were analyzed by immunoblot using the indicated antibodies. Histograms show relative protein levels of PLD1 and PLD2, which are normalized to the corresponding α-tubulin values (right panel). (B) Paraffin sections of colon tissues were subjected to immunofluorescence analyses using anti-β-catenin (Alexa fluor 488; green) and PLD (Alexa fluor 555; red) antibody. Tissues were monitored using Zeiss LSM 510 confocal microscope. Microscopy fields were observed at × 650 magnification. Data are representative of three independent experiments.

To further strengthen our results, we investigated the effect of blockade of GSK3β on PLD expression *in vivo*. LiCl was injected into mice, and expression of PLD was investigated in several tissues using anti-PLD antibody which recognizes both PLD1 and PLD2. We have reported on the specificity of the antibody to PLD [18], [30]. As analyzed by immunoprecipitation and immunoblot, LiCl increased protein levels of both PLD1 and PLD2 in colon, stomach, and liver tissues (Figure 2A). As a positive control, the level of β-catenin and NOS2, as well as phosphorylation of GSK3β, was increased in LiCl-injected tissues. A complementary result with regard to expression of PLDs following application of LiCl was also obtained by immunofluorescence in colon tissues (Figure 2B). Nuclei were counterstained by DAPI. Whereas β-catenin showed a membraneous staining pattern in PBS-injected control colon, β-catenin was increased by LiCl in the cytoplasm and nucleus of cells. PLD level was concomitantly increased in both the cytoplasm and nucleus of cells in LiCl-injected colon as β-catenin was increased (Figure 2B). Collectively, these observations indicate that expression of both PLD1 and PLD2 is enhanced in tissues of LiCl-treated mice.

### β-catenin and TCF-4 upregulate expression of PLD isozymes

**Figure 3:**
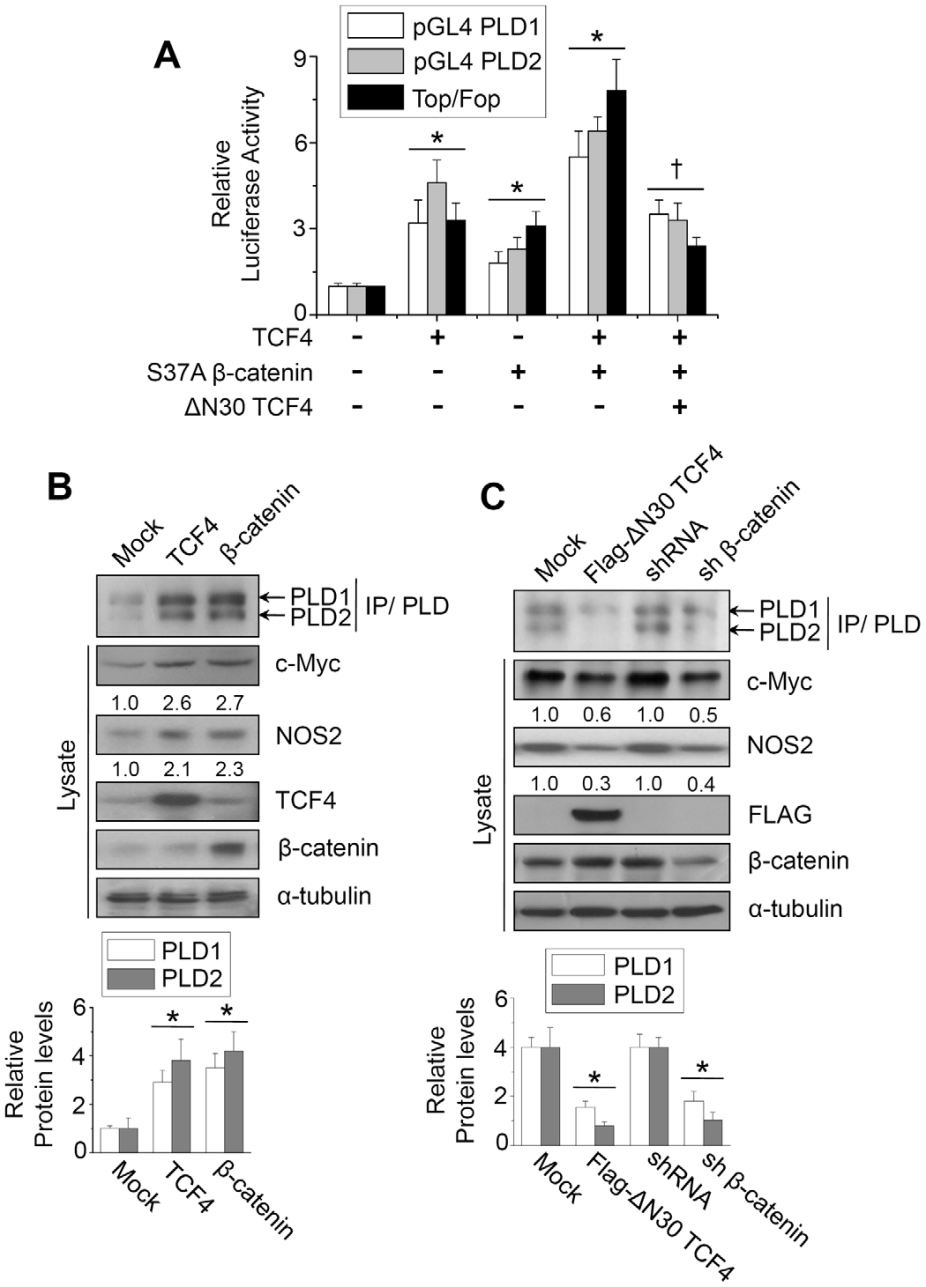
β-catenin and TCF-4 enhance the promoter activities and protein levels of PLD isozymes. (A) HCT116 cells were co-transfected with pGL4-PLD or TOP/FOP reporters and the indicated expression vectors. *P<0.01 *versus* mock; †*P*<0.05 *versus* S37A β-catenin/TCF-4. (B) Cells were transfected with either the TCF-4 or β-catenin expression vector, and lysates were immunoprecipitated or immunoblotted with the indicated antibodies (upper panel). Histograms show relative protein levels of PLD1 and PLD2, which are normalized to the corresponding α-tubulin values (lower panel). (C) Cells were transfected with ΔN30 TCF-4 or shRNA for β-catenin. Lysates were analyzed by immunoprecipitation or Western blot using the indicated antibodies (upper panel). Protein expression was quantitated by densitometer analysis. Histograms show relative protein levels of PLD1 and PLD2, which are normalized to the corresponding α-tubulin values (lower panel). Data are representative of three independent experiments.

We examined the question of whether or not expression of PLD isozymes is indeed transcriptionally activated by β-catenin or TCF-4. As shown in Figure 3*A*, ectopic expression of TCF-4 and a stable β-catenin mutant (S37A β-catenin), which is insensitive to ubiquitination of β-catenin, enhanced transcriptional activation of both PLD1 and PLD2. TCF-4 and stable β-catenin mutant significantly increased gene expression from a TCF/LEF specific luciferase reporter plasmid used as a control. This induction was significantly decreased by addition of a dominant negative (dn) TCF-4 expression vector (ΔN30 TCF-4) (Figure 3A). Moreover, analysis by immunoprecipitation and immunoblot showed that ectopic expression of β-catenin or TCF-4 increased endogenous protein levels of PLD1 and PLD2 isozymes in HCT116 cells. TCF-regulated genes, including c-Myc and NOS2, were also increased by expression of β-catenin or TCF-4 (Figure 3B). Furthermore, transfection of dnTCF-4 or depletion of β-catenin using shRNA decreased the protein levels of both PLD isozymes and Wnt target genes in HCT116 cells (Figure 3C). These results indicate that expression of PLD isozymes is upregulated by both β-catenin and TCF-4.

### β-catenin and TCF-4 bind to the TBEs of the PLD2 promoter and enhance PLD2 expression

A polymorphism of the PLD2 gene has recently been associated with prevalence of colorectal carcinoma [31]. Moreover, expression levels of PLD2 detected by real-time PCR using 97 colorectal carcinoma tissues were significantly correlated with tumor size and survival of patients with colorectal carcinoma; thus, it was suggested that PLD2 expression level could be a prognostic indicator in colorectal carcinoma [10]. Therefore, we attempted to examine the regions that are responsible for Wnt/β-catenin/TCF-4-induced PLD2 expression.

**Figure 4:**
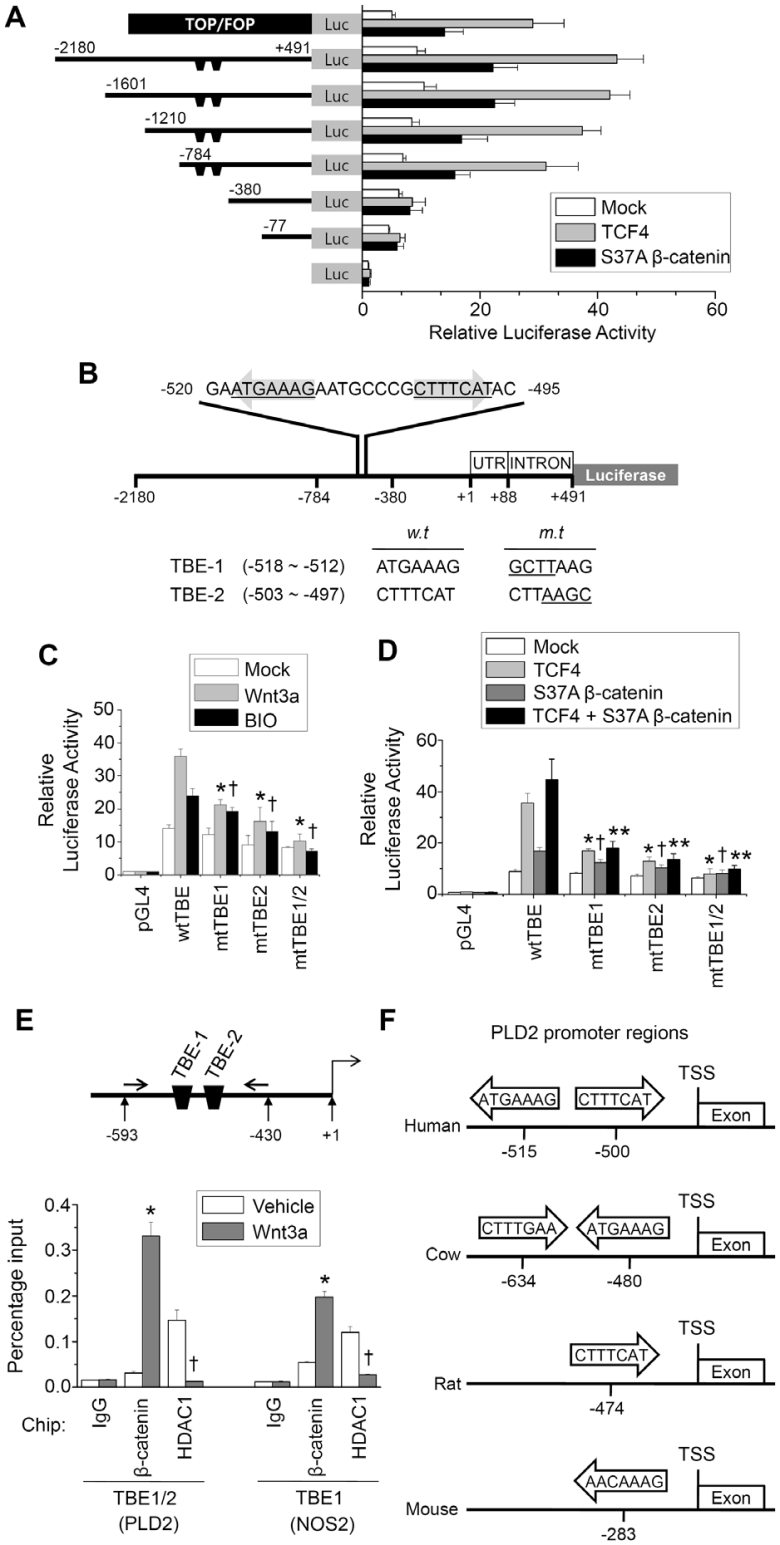
β-catenin/TCF-4 specifically binds to the TBEs of the PLD2 promoter and enhances PLD2 expression. (A) Deletion analysis of pGL4-PLD2 in HCT116 cells. A schematic representation of pGL4-PLD2 reporter constructs is shown. Cells were cotransfected with pGL4-PLD2 and the indicated expression vectors, followed by determination of luciferase activity. (B) Diagrammatic representation of the −2180 to +491 region of the human PLD2 promoter. Numbers above the lines refer to the transcription start site of the *PLD2* gene (+1). Two putative binding sites for TCF-4 are indicated on the sequence (the arrows indicate the direction). (C) HCT 116 cells were transfected with the luciferase reporter plasmid containing the wild type (wt) PLD2 promoter, one or double TBE mutant forms (mt) of PLD2 promoter, and treated with Wnt3a (150 ng/ml) or BIO (1 µM). Luciferase activities were measured. *P<0.05 *versus* wtTBE/Wnt3a; †*P*<0.01 *versus* wtTBE/BIO. (D) Cells were co-transfected with the indicated expression vectors, along with the wt or TBE mutant forms of the PLD2 promoter. Luciferase activities were measured. *P<0.05 *versus* transfected with wtTBE/β-catenin; †*P*<0.05 *versus* wtTBE/TCF4; **P<0.05 *versus* transfected with wtTBE/β-catenin/TCF4. (E) Arrows indicate position of primers used in the ChIP experiment. The ChIP assay was performed using preimmune IgG, anti-β-catenin, or anti-HDAC1 antibody and analyzed by Q-RT-PCR. As a positive control, ChIP analysis of the NOS2 promoter containing TBE was performed. *P<0.05 *versus* β-catenin/vehicle; †*P*<0.05 *versus* HDAC1/vehicle. Data are representative of four independent experiments. (F) Schematic diagram for comparison of TBEs on PLD2 promoter regions from various species. TCF-4 binding elements in 5′ flanking regions of the human PLD2 transcriptional start site (TSS) were compared with those of the genomes from 4 different species. A core *motif*, CTTTG(A/T)(A/T) [or the complementary sequence (A/T)(A/T)CAAAG] of TCF binding sequences on PLD2 promoter is highly *conserved* across species.

As shown in Figure 4A, the −2180/+491 PLD2 promoter was transactivated by TCF-4 (4.6-fold) or S37A β-catenin (2.3-fold) proteins. Sequential 5′ deletions of the PLD2 promoter to positions −1601, −1210, and −784 did not affect TCF-4- or S37A β-catenin-mediated transactivation of the PLD2 promoter (TCF-4, 4.2-fold; β-catenin 2.2-fold activation of all mutants); however, deletion of -380 regions significantly decreased TCF-4 or S37A β-catenin-stimulated PLD promoter activity. Thus, it is suggested that putative TCF binding element (TBE) at positions −784 ∼ −380 may be essential for PLD2 gene regulation by TCF-4 and β-catenin.

As shown in Figure 4B, sequence analysis of the 5′-flanking sequence of the PLD2 gene identified two putative TBEs (designated as TBE1 and TBE2). TBE1 (ATGAAAG) is located −512 b upstream, and contains a nearly inverted match with the consensus CTTTG(A/T)(A/T) sequence for TCF-4 binding [32]; TBE2 (CTTTCAT) was located −497 b upstream and nearly matched the consensus sequence (Table S2) [33].

To examine the functional importance of the TBE motif in regulation of PLD2 gene transcription, site-directed mutation of TBE sites was generated in the context of a PLD2 promoter. Mutation in the TBE1 or TBE2 site significantly decreased Wnt3a-induced PLD2 promoter activity in HCT116 cells (Figure 4C). Mutations of both the TBE1 and TBE2 sites (TBE1/2) dramatically suppressed Wnt3a-stimulated PLD2 promoter activity. Moreover, TCF-4 and/or β-catenin-induced PLD2 promoter activity was also abolished when TBE1 and TBE2 were mutated (Figure 4D). These data suggest that the PLD2 promoter is a target of the TCF/β-catenin complex via its consensus TBE.

Furthermore, we then performed a chromatin immunoprecipitation (ChIP) assay in HCT116 cells to confirm *in vivo* binding of β-catenin/TCF-4 to the PLD2 promoter. DNA-β-catenin/TCF-4 complexes were immunoprecipitated with antibodies shown in Figure 4E, followed by reversal of cross-linking and Q-RT-PCR using primers flanking both TBE1 and TBE2, which are located in proximal position to each other. As shown in Figure 4E, purified recombinant Wnt3a enhanced binding of β-catenin/TCF-4 to both TBEs of the PLD2 promoter. These results are comparable with those of the promoter assay using mutagenesis. Wnt signaling targets β-catenin to chromatin for removal of the corepressor HDAC1 (histone deacetylase 1) [34]. As expected, Wnt3a significantly suppressed binding of β-catenin to two TBEs of the PLD2 promoter after the ChIP assay, using anti-HDAC1 antibody. Moreover, we found that TCF binding sites on the PLD2 promoter are conserved across species (Figure 4F). Taken together, these data demonstrate that the PLD2 gene is a direct transcriptional target of β-catenin/TCF signaling in vivo.

### PLD isozymes are required for formation of the β-catenin/TCF-4 complex and promotion of β-catenin/TCF transcriptional activity

**Figure 5:**
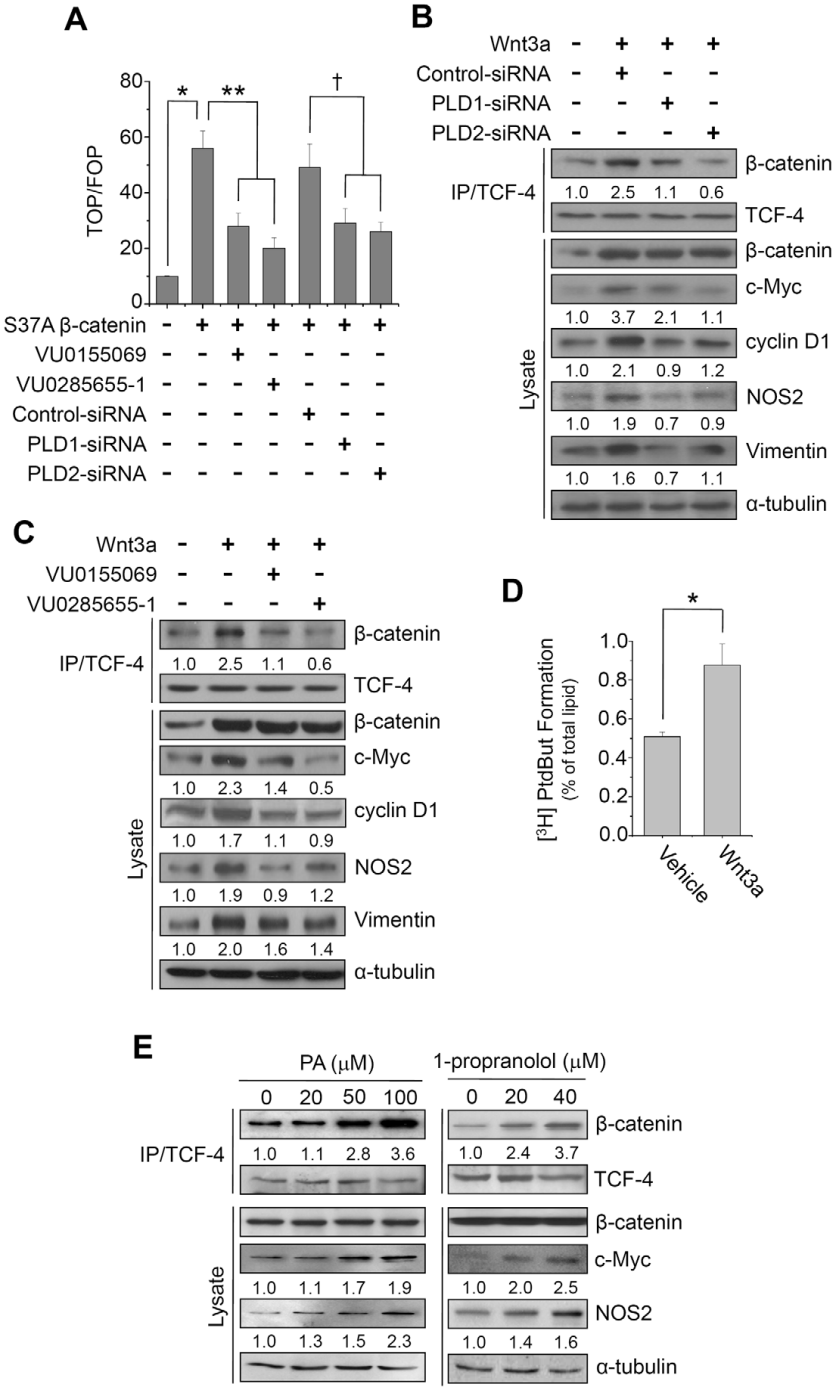
PLD isozyme is required for formation of the β-catenin/TCF-4 complex and promotion of β-catenin/TCF transcriptional activity. (A) After HCT116 cells were co-transfected with TOP/FOP reporters, S37A β-catenin, or siRNA for PLDs, cells were treated with or without the indicated dose of VU0155069 or VU0285655-1; TCF activity was then determined. *P<0.05 *versus* Mock; **P<0.05 *versus* S37A β-catenin; †*P*<0.05 *versus* S37A β-catenin-siRNA. HCT116 cells were transfected with siRNAs for PLD (B) or pretreated with 10 μM of VU0155069 or VU0285655-1 (C), and then stimulated with Wnt3a (150 ng/ml) for 20 h. (D) HCT116 cells were labeled with [^3^H] myristate and treated with Wnt3a (150 ng/ml) for 12 h. PLD activity was measured as dsescribed in Materials and Methods. *P<0.05 *versus* vehicle. (E) HCT116 cells were treated with the indicated dose of PA or 1-propranolol for 24 h. Association of TCF-4 with β-catenin was analyzed by immunoprecipitation and immunoblot using the indicated antibodies. Protein levels were determined by immunoprecipitation or immunoblotting using the indicated antibodies. Interaction levels or protein expression were quantitated by densitometer analysis. Data are representative of three independent experiments.

We investigated the question of whether or not Wnt signaling-induced PLDs upregulation might modulate β-catenin-dependent TCF transcriptional activity via a positive feedback loop. Ectopic expression of a constitutive active mutant of β-catenin increased TCF transcriptional activity in HCT116 colon cancer cells (Figure 5A). Selective PLD inhibitors have recently been developed [35], [36]. Thus, we examined the effect of PLD isoform-selective inhibitor on TCF activity. Interestingly, VU0155069, a selective PLD1 inhibitor and VU0285655-1, a selective PLD2 inhibitor [35], abolished β-catenin/TCF-4 activity (Figure 5A). Moreover, depletion of PLD1 or PLD2 using siRNA abolished Wnt3a-induced β-catenin/TCF transcriptional activation (Figure 5A). Reduction of PLD isozyme by siRNAs was analyzed by Q-RT-PCR and immunoprecipitation/immunoblot (Figure S4). Since the integrity of the β-catenin/TCF complex is required for proper transcriptional activity, we examined the possibility that Wnt-induced PLDs upregulation could enhance the association of β-catenin with TCF-4. In Wnt3a-treated cells, a substantial level of β-catenin was associated with TCF-4, while, in contrast, depletion of PLD1 or PLD2 isozyme significantly disrupted Wnt-induced β-catenin/TCF-4 association, without detectable modulation of β-catenin and TCF-4 levels (Figure 5B). Moreover, depletion of PLD1 or 2 blocked expression of Wnt3a-induced target genes, including c-Myc, Cyclin D1, NOS2, and vimentin (Figure 5B). Pretreatment with PLD1 or PLD2 selective inhibitor also decreased Wnt3a-induced β-catenin/TCF-4 interaction and Wnt3a target genes (Figure 5C), suggesting that PLD activity is required for these effects. We also found that treatment with Wnt3a (150 ng/ml) for 20 h stimulated PLD activity (Figure 5D). This activity dependency was further confirmed by treatment of cell-permeable dioctanoyl-PA and accumulation of endogenous PA by treatment of 1-propranolol, a PA phosphatase inhibitor (Figure 5E). To further confirm the involvement of PLD activity in the interaction, HCT116 cells were pretreated with either 1-butanol or 3-butanol, and then stimulated with Wnt3a. 1-Butanol was used to block PA production by PLD by virtue of phosphatidylbutanol formation through the transphosphatidylation reaction. 1-butanol, but not 3-butanol, an inactive analogue for PLD-mediated PA formation, significantly disrupted Wnt-induced β-catenin/TCF-4 association and expression of Wnt target genes without detectable modulation of β-catenin and TCF-4 levels (Figure S5). These results suggest that Wnt-induced PLD upregulation is required for β-catenin/TCF-4 transcriptional activation by increasing formation of the β-catenin/TCF-4 complex.

### PLD isozymes mediate anchorage-independent growth, migration, and invasion in concert with the Wnt/β-catenin/TCF-dependent pathway

**Figure 6:**
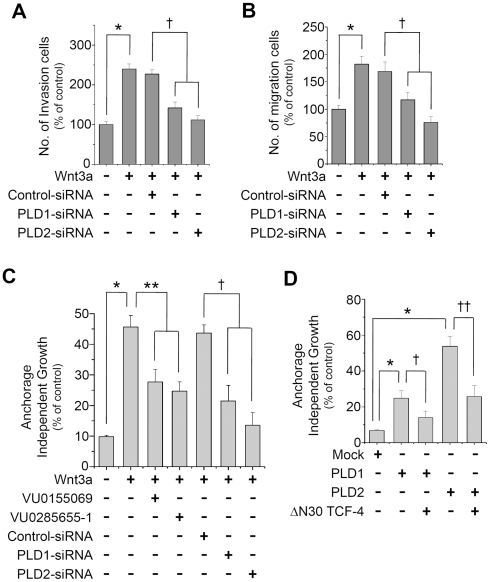
PLD isozyme mediates anchorage-independent growth, migration, and invasion in concert with the Wnt/β-catenin/TCF-dependent pathway. (A–B) HCT116 cells were transfected with or without siRNA for *PLD1* or *PLD2*, and then seeded in Matrigel-coated invasion chambers or migration chambers and stimulated with purified recombinant Wnt3a (150 ng/ml) for 24 h. The extent of invasion (A) and migration (B) were expressed as an average number of cells per microscopic field. *P<0.01 *versus* vehicle; †*P*<0.05 *versus* Wnt3a/control-siRNA. (C) HCT116 cells were transfected with siRNA for PLD1 or PLD2, suspended in agar matrix, and treated with or without 10 µM of VU0155069 or VU0285655-1 and treated with Wnt3a (150 ng/ml). Following 10 days incubation, the anchorage-independent growth assay was performed as described in the Materials and Methods section. *P<0.05 *versus* Mock; **P<0.05 *versus* Wnt3a; †*P*<0.05 *versus* Wnt3a/control-siRNA. (D) PLD1 or PLD2 mediates anchorage-independent growth via Wnt signaling. HCT116 cells were transfected with the indicated expression vectors and an anchorage-independent growth assay was performed. *P<0.05 *versus* Mock; †*P*<0.05 *versus* PLD1; ††*P*<0.05 *versus* PLD2. Data are representative of three independent experiments.

Next, we examined the question of whether or not PLD isozymes play a role in determining the motile, invasive, and tumorigenic capacity of human colon cancer cells. Stimulation of recombinant Wnt3a increased migration and invasion of HCT116 cells. Depletion of PLD1 or 2 suppressed Wnt3a-induced migratory and invasive activity (Figure 6A and B). Transfection of control siRNA had no effect. Using Q-RT-PCR, we observed reduction of PLD isozyme by siRNAs (Figure S4). These data suggest that the effect of Wnt signaling on cell migration and invasion is at least mediated via PLD isozymes. Using an *in vitro* tumor sensitivity assay, we further investigated the question of whether or not PLD isozyme is required for Wnt/β-catenin-mediated tumorigenic effects. Treatment with Wnt3a in HCT116 cells increased anchorage-independent colony growth, whereas depletion and selective inhibitor of the PLD isoform significantly suppressed Wnt3a-driven anchorage-independent growth (Figure 6C). We also found that S37A β-catenin-induced anchorage-independent growth was abolished by PLD depletion and PLD isoform selective inhibitor (data not shown). Additionally, ectopic expression of PLD1 or PLD2 enhanced anchorage-independent colony growth, which was significantly abolished by dominant negative TCF-4 (ΔN30 TCF-4), indicating that Wnt signaling is required for a PLD-induced tumorigenic effect (Figure 6D). These results suggest that PLD isozyme contributes to the anchorage-independent tumorigenic effect in concert with the Wnt/β-catenin/TCF-mediated pathway.

## Discussion

In the present study, we demonstrate that PLD isozyme is a novel downstream target and positive feedback regulator of the Wnt/β-catenin signaling pathway. Activity of β-catenin and TCF-4, key components of the Wnt signaling pathway, is frequently deregulated in colon cancers, resulting in activation of genes whose dysregulation has significant consequences on tumor development. Therefore, identification of the target genes of Wnt signaling is important for understanding β-catenin-mediated carcinogenesis. Identification of PLDs as targets of the β-catenin/TCF-4 complex further emphasizes the importance and complexity of the Wnt pathway in physiologic and pathologic processes.

Exploration of the potential roles of PLD isoforms in tumor biology has only just begun. We have reported that stable overexpression of either PLD1 or PLD2 in fibroblasts causes transformation and enhancement of tumor formation [18]. In another study, overexpression of PLD2 resulted in transformation of fibroblasts overexpressing the epidermal growth factor receptor or c-Src [37]. Elevated expression of PLD1 and PLD2 has been reported in colorectal cancer tissues [9]. One interesting report indicates that a polymorphism in the PLD2 gene is associated with increased risk of colorectal cancer [31]. Findings from a recent study showed a significant association of PLD2 expression level with tumor size (P<0.05) and survival of patients with colorectal carcinoma (P<0.05) [10]. Moreover, cells overexpressing PLD isozyme enhance tumor cell invasion and form metastases in syngeneic mice [12], [13]. The PLD2 point mutation has been found in breast cancer cells [11], and PLD2 overexpression confers a survival signal attributed to an increase in basal mTOR activity [38]. We recently reported on statistical correlation of PLD levels and β-catenin in clinical samples, as analyzed by immunohistochemistry using tissue microarray [14].

In the present study, Wnt3a and mimicking of Wnt signaling through LiCl and BIO resulted in induction of both PLD1 and PLD2 in a variety of cancer cells. Moreover, LiCl induced expression of PLD isozymes in several tissues from mice, suggesting its physiological relevance *in vivo*.

Transfection of dominant negative TCF-4 or shRNA to β-catenin abrogated endogenous PLD1 and PLD2 protein expression, which further confirms the specificity of this pathway in regulation of PLDs. Transactivation of genes by the β-catenin/TCF-4 complex involves its binding to a consensus TBE in the promoter region of the target genes [32]. Using site-directed mutagenesis and the ChIP assay, we were able to define two functional TCF binding sites on the PLD2 promoter, suggesting that the PLD2 gene is a direct transcriptional target of β-catenin/TCF signaling. Identification of active TBE sites in the present study further expands understanding of the critically important mechanisms in place for regulation of PLD2 transcription. Recently, Firestein et al. [15] have identified genes that both modulate β-catenin activity and are essential for colon cancer cell proliferation using two RNAi-based loss-of-function screens. Among one of the genes identified in this screen was PLD1. Moreover, we also found that TCF binding elements existed in the promoter region of *PLD1* gene and PLD1 also promotes Wnt signaling through TCF4 transcriptional activation.

Other signaling cascades can also impinge on expression of PLD. For instance, tumor promoters, such as phorbol myristate, stimulate selective PLD1 expression via the NFκB signaling pathway [14]. Ewing's sarcoma fusion protein, EWS/Fli, has been reported to induce selective PLD2 expression by binding to the ETS domain of the PLD2 promoter [39]. Therefore, it is likely that several signaling cascades can therefore contribute to control of PLD gene expression, which might be used to a differing extent in distinct tumor types.

Being an enzyme, PLD activity can be tightly regulated by different ways: by PKC, Rho GTPases, PIP_2_ etc. That is why the transcriptional and translational regulation of the PLD expression may be not so important for activity of the enzyme. But post-transcriptional regulation, i.e. regulation of the PLD activity may be more crucial for the cells, considering some potential therapeutical interventions using targeting PLD. Since elevation of PLD expression contribute to increase of its enzymatic activity, studies for regulation of PLD expression are also important. Interestingly, our data demonstrate that Wnt-induced PLDs upregulation promotes β-catenin/TCF transcriptional activity by increasing formation of the β-catenin/TCF-4 protein complex, and then enhances expression of TCF-4-dependent genes. PLD activity was required to increase formation of the complex and Wnt3a target gene expression.

We have recently reported on statistical correlation of PLD and β-catenin levels in clinic samples, as analyzed by immunohistochemistry using tissue microarray [14]. Co-overexpression of PLD and β-catenin was detected in 64 (52%) of 122 colorectal cancers [14], indicating *in vivo* presence of the Wnt-β-catenin-PLD positive feedback loop.

PLD protein itself does not appear to directly regulate the interaction between β-catenin and TCF-4. PLD activity might modulate the expression level of protein, which can affect association of β-catenin with TCF and β-catenin/TCF-4 activity. Several proteins such as Chibby, Groucho, FOXO3a or Plakoglobin that interactively are known to inhibit binding of β-catenin to TCF [40]–[43], might be negatively regulated by PLD1. In addition, it could not also be excluded the possibility that Wnt-induced PLD upregulation may induce a protein(s) that somehow stimulates formation of a transcriptionally active complex between TCF-4 and β-catenin. It has recently been reported that glycophosphatidylinositol (GPI)-specific PLD can promote Wnt signaling by relieving the retention of Wnt in the endoplasmic reticulum (ER) [44]. Since PC-specific PLD described in our study cannot hydrolyze GPI structure, it is not known whether PC-PLD would directly regulate Wnt signaling by relieving the retention of Wnt in the ER. However, relieving the retention of Wnt in the ER by PLD could be another mechanism by which PLD modulates Wnt signaling. Further study is needed for identification of mechanisms of PLD-mediated Wnt signaling or β-catenin/TCF interaction in other aspects.

Many of the components of Wnt/β-catenin signaling that have been studied may serve as potential targets for therapeutic agents, and blockade of Wnt/β-catenin signaling may lead to new treatment strategies. For example, chemopreventive agents, such as nonsteroidal anti-inflammatory drugs and curcumin, have been reported to downregulate β-catenin/TCF signaling [45]. A PLD inhibitor that suppresses binding of TCF-4 to β-catenin could be very valuable as a new chemopreventive drug in treatment of certain forms of cancer.

Activation of the Wnt/β-catenin pathway can result in changes in epithelial cell morphology (eg, epithelial-mesenchymal transition) and can enhance proliferation, migration, and invasion [46]–[48]. We show here that such effects on motility and invasion of Wnt/β-catenin signaling in colon carcinoma cells are mediated by PLD isozymes. Our data suggest that PLD activity is required for tumorigenic effect, such as anchorage-independent growth in concert with the Wnt/β-catenin/TCF-mediated pathway. We recently reported that PLD enhances expression of the MMP-2 gene by increasing the DNA binding activity of NFκB and Sp1, and then promotes glioma cell invasion [13]. Thus, it is speculated that PLDs can promote tumour phenotype independence of TCF-induced genes.

The present finding describing PLD isozyme as a new target and positive regulator of Wnt/β-catenin further expands understanding of the existing and rather extensive regulatory network of the Wnt signaling pathway. Accordingly, therapeutic interventions that target the PLD enzymatic activity using PLD inhibitor may be of clinical value in colorectal cancer. Future studies are warranted for further examination of the role of the Wnt/β-catenin/PLD pathway in cancer development.

## Supporting Information

Figure S1 Wnt3a increases in a time dependent manner mRNA levels of PLD isozymes in HCT116 cells. The purified recombinant Wnt3a (150 ng/ml) was treated in HCT116 cells for the indicated times, and the expression level of PLD isozymes were analyzed by Q-RT-PCR. *P<0.05 compared with non-treatment. Data represent the mean ± S.D. of three independent experiments.
(0.03 MB PDF)

Figure S2 Wnt-dependent increase of PLD mRNA is due to elevated transcription. HCT116 cells were pretreated with actinomycin D (5 µg/ml) and treated with Wnt3a (150 ng/ml) for the indicated times and then PLD mRNA levels were analyzed by Q-RT-PCR. *P<0.05 compared with non-treatment; **P<0.05 compared with Wnt3a. Data represent the mean ± S.D. of three independent experiments.
(0.04 MB PDF)

Figure S3 Wnt3a increases mRNA levels of PLD isozymes in a variety of cancer cells. HCA-7, Colo-741, RKO and HS578T cells were stimulated by Wnt3a (150 ng/ml) for 12 h. Expression of PLD isozymes were analyzed by Q-RT-PCR. *P<0.05 versus vehicle. Data represent the mean ± S.D. of three independent experiments.
(0.04 MB PDF)

Figure S4 Effect of PLD siRNAs on expression of PLD isozymes. HCT116 cells were transfected with siRNAs for control or PLD isozyme and the expression of PLD isozymes was analyzed by Q-RT-PCR and immunoprecipitation/immunoblotting using antibody to PLD. *P<0.05 versus control-siRNA.
(0.08 MB PDF)

Figure S5 PLD activity is required for Wnt-induced β-catenin/TCF-4 association. HCT116 cells were pretreated with 1- or 3-butanol (0.6%) and stimulated with Wnt3a (150 ng/ml) for 24 h. Association of TCF-4 with β-catenin was analyzed by immunoprecipitation and immunoblot using the indicated antibodies. Protein levels were determined by immunoprecipitation or immunoblotting using the indicated antibodies. Interaction levels or protein expression were quantitated by densitometer analysis. Data are representative of three independent experiments.
(0.08 MB PDF)

Table S1 Primer sets for deletion constructs of the hPLD2 promoter region.
(0.03 MB DOC)

Table S2 Consensus TBE in the PLD2 promoter.
(0.04 MB DOC)

Table S3 Primer sets for Q-RT-PCR.
(0.04 MB DOC)

Table S4 Primer sets for ChIP assay.
(0.03 MB DOC)

## References

[1] Korinek V, Barker N, Morin PJ, van Wichen D, de Weger R (1997). Constitutive transcriptional activation by a beta-catenin-Tcf complex in APC-/- colon carcinoma.. Science.

[2] Morin PJ (1999). β-Catenin signaling and cancer.. Bioassays.

[3] Morin PJ, Sparks AB, Korinek V, Barker N, Clevers H (1997). Activation of beta-catenin-Tcf signaling in colon cancer by mutations in beta-catenin or APC.. Science.

[4] Aberle H, Bauer A, Stappert J, Kispert A, Kemler R (1997). β-catenin is a target for the ubiquitin-proteasome pathway.. EMBO J.

[5] Hsu SC, Galceran J, Grosschedl R (1998). Modulation of transcriptional regulation by LEF-1 in response to Wnt-1 signaling and association with beta-catenin.. Mol Cell Biol.

[6] van Es JH, Barker N, Clevers H (2003). You Wnt some, you lose some: oncogenes in the Wnt signaling pathway.. Curr Opin Genet Dev.

[7] Exton JH (2002). Phospholipase D-structure, regulation and function. Rev. Physiol.. Biochem Pharmacol.

[8] Foster DA, Xu L (2003). Phospholipase D in Cell Proliferation and Cancer.. Mol Cancer Res.

[9] Buchanan FG, McReynolds M, Couvillon A, Kam Y, Holla VR (2005). Requirement of phospholipase D1 activity in H-RasV12-induced transformation.. Proc Natl Acad Sci U S A.

[10] Saito M, Iwadate M, Higashimoto M, Ono K, Takebayashi Y (2007). Expression of phospholipase D2 in human colorectal carcinoma.. Oncol Rep.

[11] Wood LD, Parsons DW, Jones S, Lin J, Sjöblom T (2007). The genomic landscrapes of human breast and colorectal cancers.. Science.

[12] Knoepp SM, Chahal MS, Xie Y, Zhang Z, Brauner DJ (2008). Effects of active and inactive phospholipase D2 on signal transduction, adhesion, migration, invasion, and metastasis in EL4 lymphoma cells.. Mol Pharmacol.

[13] Park MH, Ahn BH, Hong YK, Min DS (2009). Overexpression of phospholipase D enhances matrix metalloproteinase-2 expression and glioma cell invasion via protein kinase C and protein kinase A/NF-kappaB/Sp1-mediated signaling pathways.. Carcinogenesis.

[14] Kang DW, Park MH, Lee YJ, Kim HS, Kwon TK (2008). Phorbol Ester Up-regulates Phospholipase D1 but Not Phospholipase D2 Expression through a PKC/Ras/ERK/NFκB-dependent Pathway and Enhances Matrix Metalloproteinase-9 Secretion in Colon Cancer Cells.. J Biol Chem.

[15] Firestein R, Bass AJ, Kim SY, Dunn IF, Silver SJ (2008). CDK8 is a colorectal cancer oncogene that regulates beta-catenin activity.. Nature.

[16] Meng XW, Heldebrant MP, Kaufmann SH (2002). Phorbol-12-myristate 13-acetate inhibits death receptor-mediated apoptosis in Jurkat cells by disrupting FADD recruitment.. J Biol Chem.

[17] Ahn BH, Kim SY, Kim EH, Choi KS, Kwon TK (2003). Transmodulation between PLD and c-Src enhances cell proliferation.. Mol Cell Biol.

[18] Min DS, Kwon TK, Park WS, Chang JS, Park SK (2001). Neoplastic transformation and tumorigenesis associated with overexpression of phospholipase D isozymes in cultured murine fibroblasts.. Carcinogenesis.

[19] Caretti G, Salsi V, Vecchi C, Imbriano C, Mantovani R (2003). Dynamic Recruitment of NF-Y and Histone Acetyltransferases on Cell-cycle Promoters.. J Biol Chem.

[20] Chan TA, Wang Z, Dang LH, Vogelstein B, Kinzler KW (2002). Targeted inactivation of CTNNB1 reveals unexpected effects of β-catenin mutation.. Proc Natl Acad Sci USA.

[21] Sekine S, Shibata T, Sakamoto M, Hirohashi S (2002). Target disruption of the mutant β-catenin gene in colon cancer cell line HCT116: preservation of its malignant phenotype.. Oncogene.

[22] Taketo MM (2004). Shutting down Wnt signal activated cancer.. Nat Genet.

[23] Stambolic V, Ruel L, Woodgett JR (1996). Lithium inhibits glycogen synthase kinase-3 activity and mimics wingless signalling in intact cells.. Curr Biol.

[24] Sato N, Meijer L, Skaltsounis L, Greengard P, Brivanlou AH (2004). Maintenance of pluripotency in human and mouse embryonic stem cells through activation of Wnt signaling by pharmacological GSK-3beta-specific inhibitor.. Nat Med.

[25] da Costa LT, He TC, Yu J, Sparks AB, Morin PJ (1999). CDX2 is mutated in a colorectal cancer with normal APC/beta-catenin signaling.. Oncogene.

[26] Schlosshauer PW, Brown SA, Eisinger K, Yan Q, Guglielminetti ER (2000). APC truncation and increased beta-catenin levels in a human breast cancer cell line.. Carcinogenesis.

[27] Suzuki H, Toyota M, Carraway H, Gabrielson E, Ohmura T (2008). Frequent epigenetic inactivation of Wnt antagonist genes in breast cancer.. Br J Cancer.

[28] Ilyas M, Tomlinson IP, Rowan A, Pignatelli M, Bodmer WF (1997). Beta-catenin mutations in cell lines established from human colorectal cancers.. Proc Natl Acad Sci U S A.

[29] Rowan AJ, Lamlum H, Ilyas M, Wheeler J, Straub J (2000). APC mutations in sporadic colorectal tumors: a mutational ‘hotspot’ and interdependence of the ‘two hits’.. Proc Natl Acad Sci U S A.

[30] Min DS, Choi JS, Chun MH, Chung JW, Lee MY (2001). Transient expression of phospholipase D1 in developing rat hippocampus.. Neurosci Lett.

[31] Yamada Y, Hamajima N, Kato T, Iwata H, Yamamura Y (2003). Association of a polymorphism of the phospholipase D2 gene with the prevalence of colorectal cancer.. J Mol Med.

[32] He TC, Sparks AB, Rago C, Hermeking H, Zawel L (1998). Identification of c-MYC as a target of the APC pathway.. Science.

[33] He TC, Chan TA, Vogelstein B, Kinzler KW (1999). PPARδ is an APC-regulated target of nonsteroidal anti-inflammatory drugs.. Cell.

[34] Li J, Wang CY (2008). TBL1-TBLR1 and beta-catenin recruit each other to Wnt target-gene promoter for transcription activation and oncogenesis.. Nat Cell Biol.

[35] Scott SA, Selvy PE, Buck JR, Cho HP, Criswell TL (2009). Design of isoform-selective phospholipase D inhibitors that modulate cancer cell invasiveness.. Nat Chem Biol.

[36] Su W, Yeku O, Olepu S, Genna A, Park JS (2009). 5-Fluoro-2-indolyl des-chlorohalopemide (FIPI), a Phospholipase D pharmacological inhibitor that alters cell spreading and inhibits chemotaxis.. Mol Pharmacol.

[37] Joseph T, Wooden R, Bryant A, Zhong M, Lu Z (2001). Transformation of cells overexpressing a tyrosine kinase by phospholipase D1 and D2.. Biochem Biophys Res Commun.

[38] Rodrik V, Zheng Y, Harrow F, Chen Y, Foster DA (2005). Survival signals generated by estrogen and phospholipase D in MCF-7 breast cancer cells are dependent on Myc.. Mol Cell Biol.

[39] Kikuchi R, Murakami M, Sobue S, Iwasaki T, Hagiwara K (2007). Ewing's sarcoma fusion protein, EWS/Fli-1 and Fli-1 protein induce PLD2 but not PLD1 gene expression by binding to an ETS domain of 5′ promoter.. Oncogene.

[40] Takemaru K, Yamaguchi S, Lee YS, Zhang Y, Carthew RW (2003). Chibby, a nuclear beta-catenin-associated antagonist of the Wnt/Wingless pathway.. Nature.

[41] Daniels DL, Weis WI (2005). Beta-catenin directly displaces Groucho/TLE repressors from Tcf/Lef in Wnt-mediated transcription activation.. Nat Struct Mol Biol.

[42] Essers MA, de Vries-Smits LM, Barker N, Polderman PE, Burgering BM (2005). Functional interaction between beta-catenin and FOXO in oxidative stress signaling.. Science.

[43] Hoogeboom D, Essers MA, Polderman PE, Voets E, Smits LM (2008). Interaction of FOXO with beta-catenin inhibits beta-catenin/T cell factor activity.. J Biol Chem.

[44] Zoltewicz JS, Ashique AM, Choe Y, Lee G, Taylor S (2009). Wnt signaling is regulated by endoplasmic reticulum retention.. PLoS ONE.

[45] Jaiswal AS, Marlow BP, Gupta N, Narayan S (2002). Beta-catenin-mediated transactivation and cell-cell adhesion pathways are important in curcumin (diferuylmethane)-induced growth arrest and apoptosis in colon cancer cells.. Oncogene.

[46] Hay ED, Zuk A (1995). Transformations between epithelium and mesenchyme: normal, pathological, and experimentally induced.. Am J Kidney Dis.

[47] Ben-Ze'ev A, Geiger B (1998). Differential molecular interactions of beta-catenin and plakoglobin in adhesion, signaling and cancer.. Curr Opin Cell Biol.

[48] Brembeck FH, Schwarz-Romond T, Bakkers J, Wilhelm S, Hammerschmidt M (2004). Essential role of BCL9-2 in the switch between beta-catenin's adhesive and transcriptional functions.. Genes Dev.

