# Supplementary figures and images for: Positive Feedback Regulation between Phospholipase D and Wnt Signaling Promotes Wnt-Driven Anchorage-Independent Growth of Colorectal Cancer Cells
Source: PLoS One. 2010 Aug 12;5(8):e12109. doi: 10.1371/journal.pone.0012109 (PMC2920823; doi:10.1371/journal.pone.0012109)

Figure S1

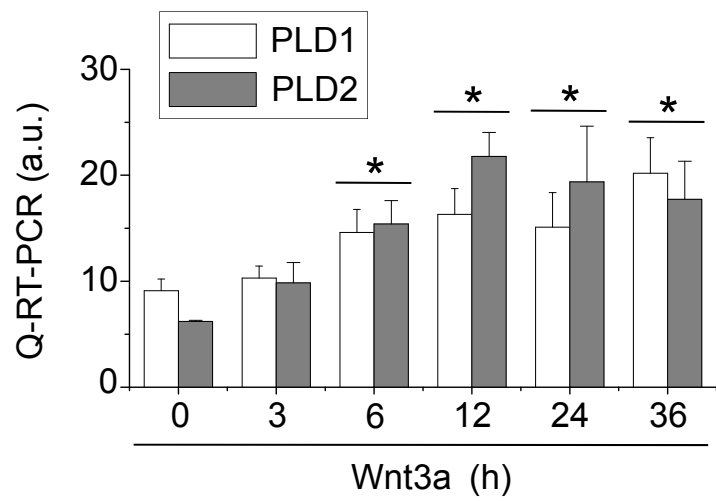

Supplement: Figure S1 — Wnt3a increases in a time dependent manner mRNA levels of PLD isozymes in HCT116 cells. The purified recombinant Wnt3a (150 ng/ml) was treated in HCT116 cells for the indicated times, and the expression level of PLD isozymes were analyzed by Q-RT-PCR. *P<0.05 compared with non-treatment. Data represent the mean ± S.D. of three independent experiments. (0.03 MB PDF) [file pone.0012109.s001.pdf]

Figure S2

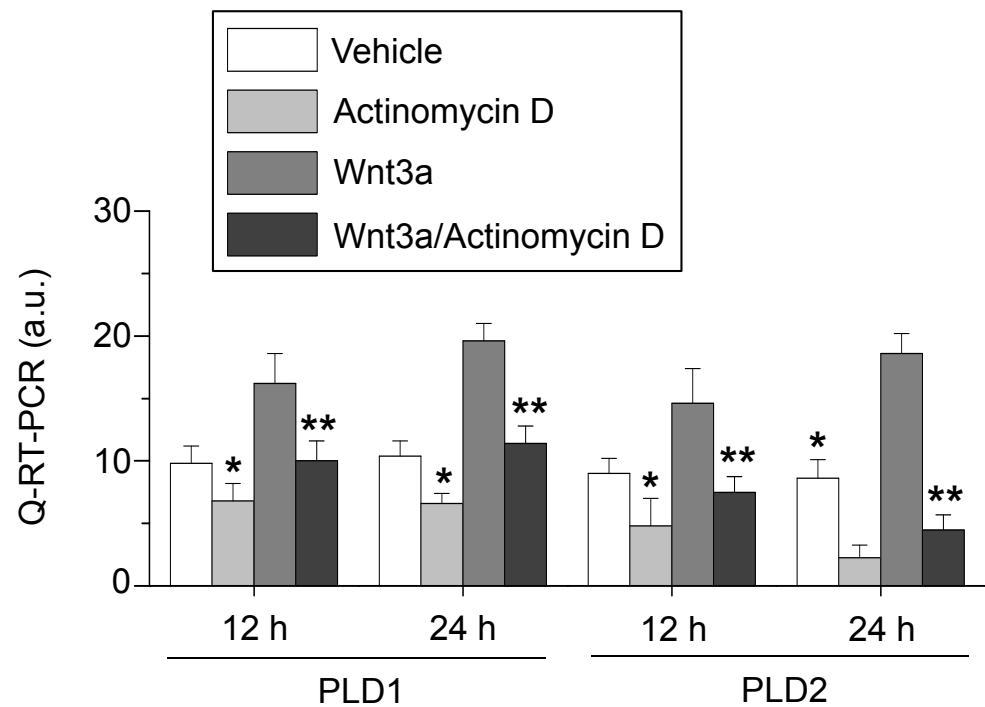

Supplement: Figure S2 — Wnt-dependent increase of PLD mRNA is due to elevated transcription. HCT116 cells were pretreated with actinomycin D (5 µg/ml) and treated with Wnt3a (150 ng/ml) for the indicated times and then PLD mRNA levels were analyzed by Q-RT-PCR. *P<0.05 compared with non-treatment; **P<0.05 compared with Wnt3a. Data represent the mean ± S.D. of three independent experiments. (0.04 MB PDF) [file pone.0012109.s002.pdf]

Figure S3

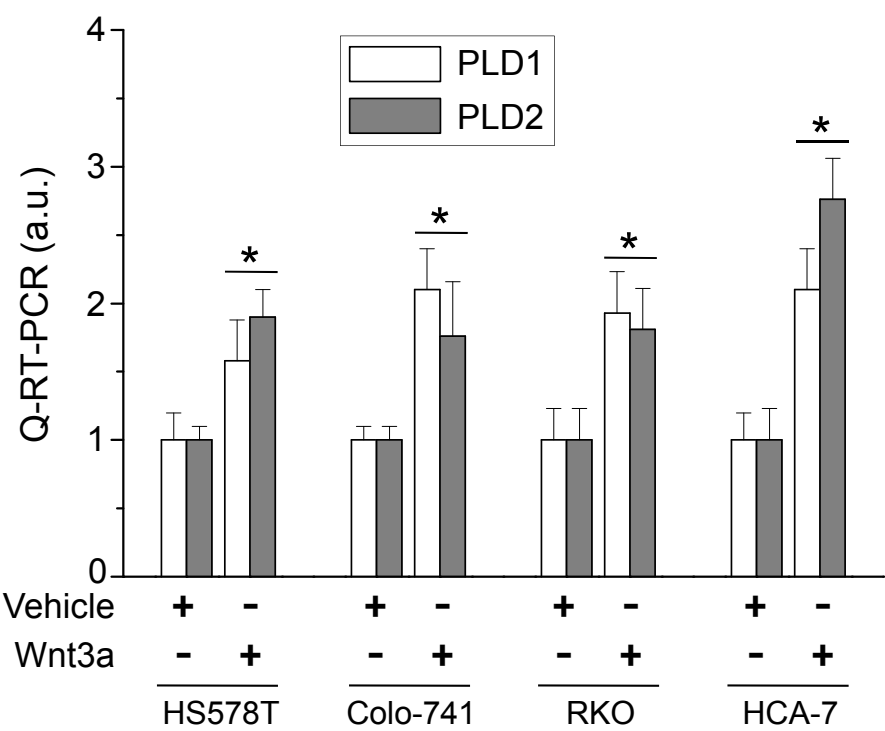

Supplement: Figure S3 — Wnt3a increases mRNA levels of PLD isozymes in a variety of cancer cells. HCA-7, Colo-741, RKO and HS578T cells were stimulated by Wnt3a (150 ng/ml) for 12 h. Expression of PLD isozymes were analyzed by Q-RT-PCR. *P<0.05 versus vehicle. Data represent the mean ± S.D. of three independent experiments. (0.04 MB PDF) [file pone.0012109.s003.pdf]

Figure S4

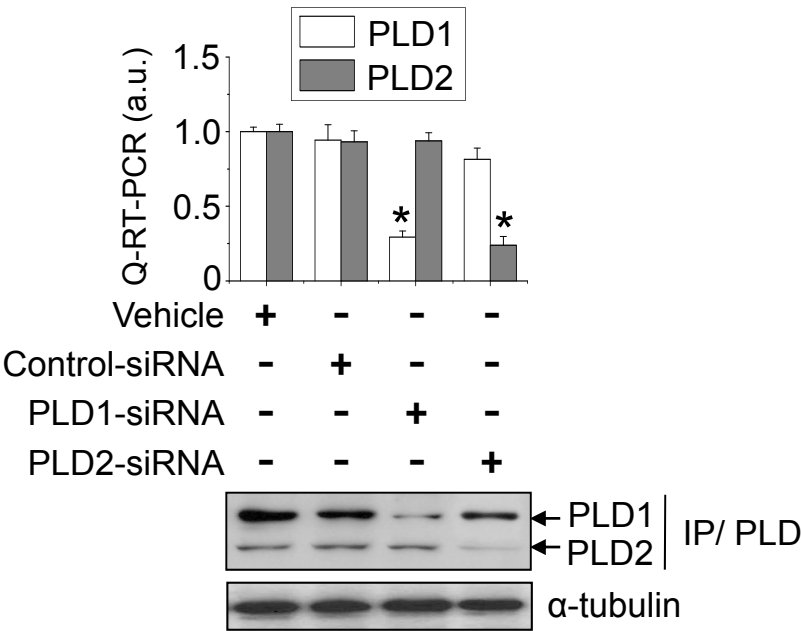

Supplement: Figure S4 — Effect of PLD siRNAs on expression of PLD isozymes. HCT116 cells were transfected with siRNAs for control or PLD isozyme and the expression of PLD isozymes was analyzed by Q-RT-PCR and immunoprecipitation/immunoblotting using antibody to PLD. *P<0.05 versus control-siRNA. (0.08 MB PDF) [file pone.0012109.s004.pdf]

Figure S5

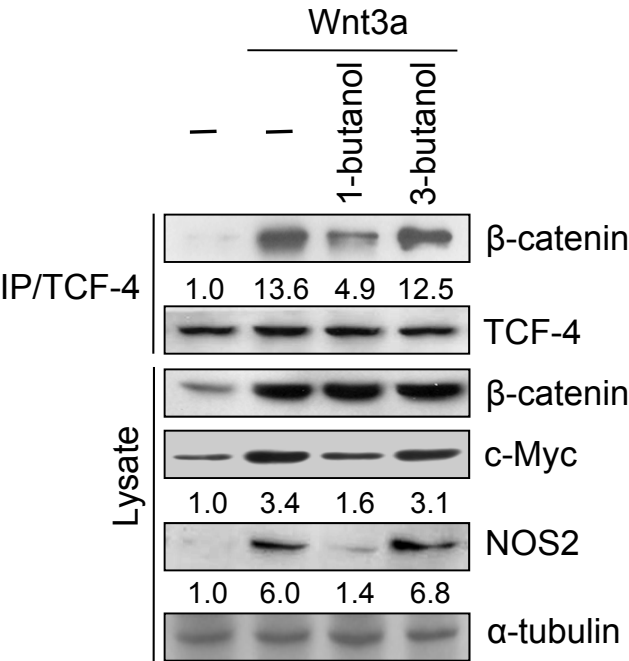

Supplement: Figure S5 — PLD activity is required for Wnt-induced β-catenin/TCF-4 association. HCT116 cells were pretreated with 1- or 3-butanol (0.6%) and stimulated with Wnt3a (150 ng/ml) for 24 h. Association of TCF-4 with β-catenin was analyzed by immunoprecipitation and immunoblot using the indicated antibodies. Protein levels were determined by immunoprecipitation or immunoblotting using the indicated antibodies. Interaction levels or protein expression were quantitated by densitometer analysis. Data are representative of three independent experiments. (0.08 MB PDF) [file pone.0012109.s005.pdf]
