# Supplementary material for: Positive Feedback Regulation between Phospholipase D and Wnt Signaling Promotes Wnt-Driven Anchorage-Independent Growth of Colorectal Cancer Cells
Source: PLoS One. 2010 Aug 12;5(8):e12109. doi: 10.1371/journal.pone.0012109 (PMC2920823; doi:10.1371/journal.pone.0012109)
Supplement: Table S1 — Primer sets for deletion constructs of the hPLD2 promoter region. (0.03 MB DOC) [file pone.0012109.s006.doc]

**Table S1.** Primer sets for deletion constructs of the hPLD2 promoter region.

| **Direction** | **Position** | **Seguence (5´ to 3´)** |
| --- | --- | --- |
| Forward | -2180 | CCG **GGT ACC** CCA AGT GTA TGC TCC TAT GCA ATC ACC |
| Forward | -1601 | CCG **GGT ACC** CCA ACA TGG TGA AAC CCC GTC |
| Forward | -1210 | CCG **GGT ACC** GGC AAC ACA GCA AGA CTC CAT CTC |
| Forward | -784 | CCG **GGT ACC** GCA AAC TTA CAG GAA TGT TTA CAG GAG |
| Forward | -380 | CCG **GGT ACC** CGC AGC GGA GGC GCG TCC |
| Forward | -77 | CCG **GGT ACC** CCC GCG GAG GTG AGG CCG |
| Reverse | +491 | GA **AGA TCT** GTT TGC AAC GCC CCA CCG ACC CCT TCG |
| Restriction enzyme sites : Bold type. | | |
