# Supplementary material for: Positive Feedback Regulation between Phospholipase D and Wnt Signaling Promotes Wnt-Driven Anchorage-Independent Growth of Colorectal Cancer Cells
Source: PLoS One. 2010 Aug 12;5(8):e12109. doi: 10.1371/journal.pone.0012109 (PMC2920823; doi:10.1371/journal.pone.0012109)
Supplement: Table S2 — Consensus TBE in the PLD2 promoter. (0.04 MB DOC) [file pone.0012109.s007.doc]

**Table S2. Consensus TBE in the PLD2 promoter.**

| **Oligo/plasmid** | **Position** | **Seguence (5´ to 3´)** |
| --- | --- | --- |
| TBE1 |  |  |
| WT | -518 to -512 | ATGTTTGGTGAATGA**ATGAAAG**AATGCCCGCTTTCA |
| MT | -518 to -512 | ATGTTTGGTGAATGA**GCTTAAG**AATGCCCGCTTTCA |
| TBE2 |  |  |
| WT | -503 to -497 | GAATGAAAGAATGCCCG**CTTTCAT**ACACTAATGGGTTTGT |
| MT | -503 to -497 | GAATGAAAGAATGCCCG**CTTAAGC**ACACTAATGGGTTTGT |
| TBE1/2 |  |  |
| TBE1-MT | -503 to -497 | GA**GCTTAAG**AATGCCCG**CTTTCAT**ACACTAATGGGTTTGT |
| TBE1/2-MT | -503 to -497 | GA**GCTTAAG**AATGCCCG**CTTAAGC**ACACTAATGGGTTTGT |
| Binding sites : Bold type. Mutations : Underline type | | |
