# Supplementary material for: Positive Feedback Regulation between Phospholipase D and Wnt Signaling Promotes Wnt-Driven Anchorage-Independent Growth of Colorectal Cancer Cells
Source: PLoS One. 2010 Aug 12;5(8):e12109. doi: 10.1371/journal.pone.0012109 (PMC2920823; doi:10.1371/journal.pone.0012109)
Supplement: Table S3 — Primer sets for Q-RT-PCR. (0.04 MB DOC) [file pone.0012109.s008.doc]

**Table S3. Primer sets for Q-RT-PCR.**

| Q-RT-PCR | Primer | Direction | Seguence (5´ to 3´) |
| --- | --- | --- | --- |
|  | PLD1 | Forward | AAGGCGGCTCGTGATGTGG |
|  |  | Reverse | ATGGGCTGTTGTTTGAGACTTTGG |
|  | PLD2 | Forward | CATCCAGGCCATTCTGCAC |
|  |  | Reverse | GTGCTTCCGCAGACTCAAGG |
|  | c-Myc | Forward | TCCAGCTTGTACCTGCAGGATCTGA |
|  |  | Reverse | CCTCCAGCAGAAGGTGATCCAGACT |
|  | NOS2 | Forward | TGCCAGATGGCAGCATCAGA |
|  |  | Reverse | TTTCCAGGCCCATTCTCCTGC |
|  | GAPDH | Forward | GTGGTCTCCTCTGACTTCAAC |
|  |  | Reverse | TCTCTTCCTCTTGTGCTCTTG |
