# Supplementary material for: Positive Feedback Regulation between Phospholipase D and Wnt Signaling Promotes Wnt-Driven Anchorage-Independent Growth of Colorectal Cancer Cells
Source: PLoS One. 2010 Aug 12;5(8):e12109. doi: 10.1371/journal.pone.0012109 (PMC2920823; doi:10.1371/journal.pone.0012109)
Supplement: Table S4 — Primer sets for ChIP assay. (0.03 MB DOC) [file pone.0012109.s009.doc]

**Table S4. Primer sets for Chip assay.**

| Promoter | Primer | Direction | Seguence (5´ to 3´) |
| --- | --- | --- | --- |
| PLD2 | TBE1/2 | Forward | ACTACCATACTCTCAATTTCTGGCAC |
|  |  | Reverse | CCTAGCCCAGATAGCGTTTGTACC |
| NOS2 | TBE1 | Forward | CAGCCTGGCATAGAAACAGATC |
|  |  | Reverse | CAAGGTCACACAGCAAACAGCC |
| PLD2 Regions : TBE1/2 (-593 to -432), NOS2 Regions : TBE1 (-4050 to -3601), | | | |
